# Postoperative thrombotic thrombocytopenic purpura after aortofemoral bypass

**DOI:** 10.3402/jchimp.v2i4.19797

**Published:** 2013-01-07

**Authors:** Naba R. Mainali, Madan R. Aryal, Madan Badal, Vijaya R. Bhatt, Richard Alweis

**Affiliations:** 1 Department of Medicine, The Reading Hospital and Medical Center, West Reading, PA, USA; 2 Department of Medicine, Division of Hematology–Oncology, University of Nebraska Medical Center, Omaha, NE, USA

**Keywords:** postoperative thrombotic thrombocytopenic purpura, aortofemoral bypass, plasmapheresis

## Abstract

Thrombotic thrombocytopenic purpura (TTP) is uncommon in the postoperative setting, even more so after vascular surgery. We present a case of thrombocytopenia after aortofemoral bypass, which highlights the importance of a high index of suspicion for postoperative TTP to avoid life-threatening consequences.

Thrombotic thrombocytopenic purpura is a life-threatening clinical syndrome characterized by thrombocytopenia and microangiopathic hemolytic anemia (MAHA). Postoperative thrombotic thrombocytopenic purpura (pTTP) is an uncommon and distinct entity that has been mainly labelled with cardiothoracic and vascular surgeries (1). Their associations with appendectomy and gynecological surgeries have also been described (1). In the postoperative period, it can be challenging to differentiate this from other causes of renal failure, anemia, and thrombocytopenia. Early recognition is crucial in order to prevent significant mortality. Active treatment with plasmapharesis while awaiting a confirmatory test result is prudent.

## Case report

A 66-year-old Caucasian male with a history of hypertension, type II diabetes mellitus, and tobacco abuse underwent an elective aortobifemoral bypass with bilateral profundoplasties and ligation of common iliac artery aneurysms. He tolerated the surgery well. Intraoperatively and postoperatively, he did not receive nephrotoxic drugs or intravenous contrast. The operative record showed that normal blood pressure was maintained throughout the surgery. The only new medication the patient received during the hospitalization was enoxaparin, administered for the prophylaxis of deep vein thrombosis. His home medications including lisinopril, ranitidine, metoprolol, lovastatin, and verapamil were continued postoperatively. His preoperative blood work including complete blood count, basic metabolic panel, liver function test, clotting studies, and chest X-ray were all within normal limits.

On postoperative day 3, he developed sudden thrombocytopenia and anemia, with platelet count decreasing from 180,000 to 38,000/ µL and hemoglobin decreasing from 15.2 to 8.3 g/dL. Physical examination remained normal with normal vital signs including normal temperature. No obvious bleeding sources could be identified. Laboratory values were also significant for acute renal failure (creatinine 4.12 mg/dL), reduced haptoglobin level of 27 mg/dL (normal range 36–195 mg/dL), elevated LDH level of 974 IU/L (normal 94–202 IU/L), increased total bilirubin of 4 mg/dL with direct of 0.3 mg/dL, and elevated CPK at 8405 IU/L. Peripheral blood smear revealed schistocytes. Liver function and coagulation profile (PT, PTT, fibrinogen, FDP) were within normal limits. HbsAg, HIV, direct and indirect Coombs test, antinuclear antibody (ANA), and antineutrophil cytoplasmic antibody (ANCA) were all negative. Urine analysis, blood culture, urine culture, sputum culture, CT scan of the abdomen, and chest X-ray were unrevealing. There was approximately 700 cc of blood loss. Other sources of anemia like gastrointestinal bleeding were ruled out by a negative hemoccult test and retroperitoneal bleeding by normal CT scan of the abdomen and pelvis. On day 4, his platelet count further decreased to 21,000/µL and creatinine continued to rise to 5.42 mg/dL. He developed oliguria despite aggressive hydration and had significant alteration in mental status. With the patient's rising serum creatinine and falling platelets, the differential diagnosis of heparin-induced thrombocytopenia (HIT) and disseminated intravascular coagulation (DIC) due to sepsis, postoperative acute tubular necrosis (ATN) due to hypovolemia and postoperative TTP were considered. However, HIT antibody was negative and since the sepsis workup was negative, DIC was unlikely. ATN from intraoperative ischemia was less likely due to the presence of normal hemodynamics during the surgery.

Hematology and nephrology consultation both stressed the high likelihood of TTP and as a result, plasmapharesis was immediately started for suspected postoperative TTP on day 4. The patient had significant recovery after 12 cycles of plasmapharesis with normalization of hemoglobin (13.5 g/dL), platelets (177,000/µL), creatinine (1.37 mg/dL), urea, and LDH over 10 days. His mental status also returned to baseline. Further tests, including Serotonin Release Assay (SRA), antihospholipid antibody (lupus anticoagulant and anticardiolipin antibodies), serum complement level, ANA, and urine eosinophils, were all negative. ADAMTS 13 activity was at 69% (normal range: 60–130%). Since the patient improved with plasmapharesis in the setting of low platelets, abnormal renal function tests and schistocytes on peripheral blood smear with the change in mental status, the diagnosis of pTTP was consistent.

## Discussion

Thrombocytopenia and anemia are very common in the postoperative period. Causes such as blood loss, hemodilution, infection, DIC or HIT are fairly common after surgery and differentiating one from the other can be very confusing. Regardless of the cause, many patients have spontaneous recovery after conservative management. It is rare for thrombocytopenia and anemia to persist without obvious causes (2) which necessitate further evaluation.

TTP is one of the dreaded but uncommon causes of thrombocytopenia and anemia. Classically, TTP is associated with certain infections, drugs (e.g., clopidogrel, mitomycin, ticlopidine, quinine), pregnancy, collagen vascular disease, and malignancies (3–5), none of which were present in our case. A clinical entity identical to classic TTP that rarely occurs postoperatively is called postoperative TTP, and it occurs usually after 4–5 days of surgery (6, 7). To our knowledge, fewer than 42 cases of pTTP have been reported in the literature. One should suspect it in a patient with normal hemoglobin preoperatively who subsequently develops MAHA and thrombocytopenia. The surgical procedures associated with pTTP include cardiac surgery, such as coronary artery bypass grafting, vascular surgery, gastrointestinal surgery, and knee replacements (3, 4, 8). As in classic TTP, fever, impaired renal function, and altered mental status are variably present and are not usually required for diagnosis. Therefore, pTTP is a diagnosis of exclusion (9–11).

The exact pathophysiology of pTTP remains unclear. It is believed that endothelial damage at the time of surgery releases high-molecular-weight von Willebrand factor (vWF) multimers. This seems to be sufficient to overwhelm available vWF-cleaving enzymes, more likely in patients with a marginal vWF-cleaving enzyme level (3, 12, 13). Schistocytes on the peripheral smear are a marker of hemolysis in TTP, although it may be absent in pTTP. Peripheral smears will also help to differentiate from the toxic granulation found in postoperative infections. Normal prothrombin time (PT), partial prothrombin time (PTT), and fibrin split products helps to differentiate this condition from DIC. Evidence of hemolysis can be assessed by reticulocyte count, serum lactate dehydrogenase, and haptoglobulin which are also useful markers of disease activity and should be followed closely. HIT antibody (antibody against platelet factor 4) helps to differentiate pTTP from HIT. The diagnostic test for pTTP is low ADAMTS 13 activity, which is fairly specific but not available in all of the centers. As in our case, a patient may have clinical TTP without severe deficiency of ADAMTS 13, and it can be severely deficient without clinical manifestations in some cases (14). Therefore, this should not delay the administration of life-saving intervention such as plasmapharesis (4, 15).

In the past, TTP was nearly uniformly fatal. However, with the availability of plasma exchange, morbidity, and mortality are low. Most patients require five to ten daily plasma exchanges. If improvement occurs, the frequency of exchange may be reduced (6, 7, 16, 17). The benefit of adjunctive treatments, such as corticosteroids, is unclear. Although platelet transfusion may be beneficial in clinically serious bleeding, it is not routinely recommended (18–21). Cases that are resistant to usual therapy are treated with immunomodulation (22) with vincristine (23, 24) cyclophosphamide (25), cyclosporine or splenectomy (26).

Because of the rarity of the condition, the diagnosis of pTTP may be missed. Moreover, the broad differential for this condition including acute kidney injury, infection, postoperative and drug-induced thrombocytopenia, HIT, and DIC may delay the timely institution of treatment. In our case, early recognition and prompt introduction of plasmapharesis saved this patient's life. Our case emphasizes the importance of this early recognition and treatment based on the presence of thrombocytopenia and MAHA in the absence of alternative causes, in order to prevent mortality from pTTP. If a patient fails to improve despite plasma exchange, another diagnosis should be sought.
